# The Different Dose-Volume Effects of Normal Tissue Complication Probability Using LASSO for Acute Small-Bowel Toxicity during Radiotherapy in Gynecological Patients with or without Prior Abdominal Surgery

**DOI:** 10.1155/2014/143020

**Published:** 2014-07-16

**Authors:** Tsair-Fwu Lee, Eng-Yen Huang

**Affiliations:** ^1^Medical Physics and Informatics Laboratory of Electronics Engineering, National Kaohsiung University of Applied Sciences, Kaohsiung 807, Taiwan; ^2^Department of Radiation Oncology, Kaohsiung Chang Gung Memorial Hospital and Chang Gung University College of Medicine, 123 Ta-Pei Road, Niao-Sung District, Kaohsiung 83305, Taiwan; ^3^School of Traditional Chinese Medicine, Chang Gung University College of Medicine, Taoyuan 333, Taiwan

## Abstract

*Purpose*. To develop normal tissue complication probability (NTCP) model with least absolute shrinkage and selection operator (LASSO) to analyze dose-volume effects that influence the incidence of acute diarrhea among gynecological patients with/without prior abdominal surgery.* Methods and Materials*. Ninety-five patients receiving gynecologic radiotherapy (RT) were enrolled. The endpoint was defined as the grade 2^+^ acute diarrhea toxicity during treatment. We obtained the range of small-bowel volume in V4 Gy to V40 Gy of dose.* Results*. The number of patients experiencing grade 2^+^ acute diarrhea toxicity was 23/61 (38%) in the group without abdominal surgery (group 0) and 17/34 (50%) patients with abdominal surgery (group 1). The most significant predictor was found for the logistic NTCP model with V16 Gy as the cutoff dose for group 0 and V40 Gy for group 1. Logistic regression NTCP model parameters were TV_10_ ≈ 290 cc for V16 Gy and TV_10_ ≈ 75 cc for V40 Gy, respectively.* Conclusion*. To keep the incidence of grade 2^+^ acute small-bowel toxicity below 10%, we suggest that small-bowel volume above the prescription dose (V16 Gy) should be held to <290 cc for patients without abdominal surgery, and the prescription dose (V40 Gy) should be maintained <75 cc for patients with abdominal surgery.

## 1. Introduction

Radiation therapy (RT) plays an important role in the treatment of abdominal and pelvic disease. However, radiation-induced acute diarrhea is common side effect which correlates with dose-volume effect of small bowel [1–3]. The acute diarrhea induced by radiotherapy includes increased stool frequency, increased degrees of hardness and volume of stool, severely watery stools, or a mixture of blood, serum, mucus, and other items. Acute diarrhea not only affects the patient's quality of life, but is also serious and requires hospitalization due to dehydration. Severe acute diarrhea has led to the failure of complete therapy or reduction in the total dose, all of which may diminish the effectiveness of therapy [4]. The relationship of the small-bowel dose and irradiative volume with acute diarrhea would make progress in RT strategies and help to direct further efforts to reduce the incidence of this intractable side effect [5].

Prior abdominal surgery may influence the incidence of acute diarrhea, as has been reported in previous studies [5–7]. The relationship between the volume of small bowel irradiated and the degree of acute small-bowel toxicity experienced has been well recognized but poorly quantified. In this work, we introduce the normal tissue complication probability (NTCP) model to quantify the relationship between the incidence of acute diarrhea and dose-volume effects of small bowel, which potentially identify a more specific dose-volume relationship.

NTCP modelling in radiation therapy aims to describe the correlation between dosimetric parameters and the probability of side effects [8–10]. We can assign the great information about inhomogeneous dose distributions and corresponding outcome data in large patient populations into few-parametric models [11]. This single probability value is more clearly able to reveal the relationship between dose volume and acute diarrhea in an individual treatment plan.

The least absolute shrinkage and selection operator (LASSO) is based on shrinkage estimated and has been widely used in the statistics field. Xu et al. [12, 13] introduced LASSO to build NTCP models of xerostomia after three-dimensional conformal radiation therapy (3D-CRT) for head and neck cancer. The advantages of LASSO include (1) a smaller mean squared error (MSE) than conventional methods; (2) handling the multicollinearity problem; (3) overall variable selection; and (4) coefficients shrink [12, 13]. In addition, being easy to implement is one of the merits that attract users. Xu et al. recommended the LASSO method for the NTCP predictive factor selection [12, 14].

Validated NTCP models of small-bowel toxicity are needed to provide evidence for dosimetric guidelines in treatment planning and protocols [15]. Previous studies have been reported about dose-volume parameters and acute gastrointestinal toxicity in various pelvic malignancies. However, NTCP models describing RT-induced acute diarrhea in gynecologic cancer, particularly taking into account patients with/without prior abdominal surgery, are lacking. Therefore, the purpose of this study was to develop a logistic regression NTCP model with LASSO to make valid predictions about the incidence of acute diarrhea among gynecological patients.

## 2. Materials and Methods

### 2.1. Patient Characteristics

The study population was composed of 95 patients receiving gynecologic radiotherapy. Patients were grouped into without (group 0) or with (group 1) prior abdominal surgery. Those patients were initially evaluated with pathologic assessment, physical examination, routine laboratory work, abdominal-pelvic CT scan, and chest X-ray. All patients were treated by a single radiation oncologist (E.Y.H.). Patients with a history of intestinal diseases such as inflammatory bowel disease, diverticulitis, and autoimmune diseases were excluded. The study was under the proof of the institutional review board of the hospital (97-1370B). Patient characteristics are summarized in Table 1.

### 2.2. Bowel Delineation

To accurately calculate irradiated small-bowel volume, patients received computed tomography simulation in the supine position, with a thermoplastic body cast for immobilization, an emptied bladder and bowel, and with small-bowel contrast (4% Gastrografin). The patients were scanned from the mid-abdomen to 10 cm below the ischial tuberosities in 0.5 cm increment slices. Therefore, the volumetric data were transferred to the Pinnacle treatment planning system (Philips, Fitchburg, WI).

**Figure 1:**
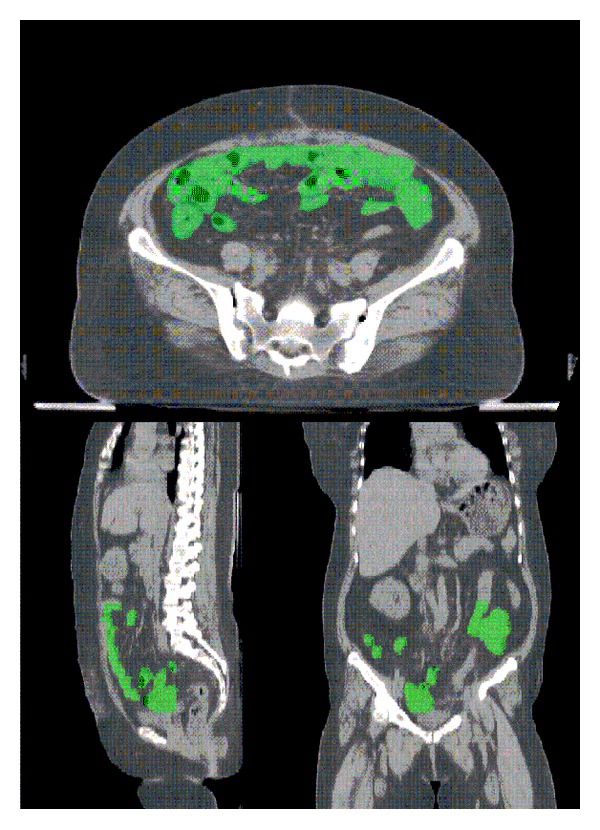
The delineation of the bowels.

The small bowel was contoured from the L4-5 interspace to its lowest extent in the pelvis. The outermost extent of the contrast-enhanced small-bowel loops was outlined on each axial CT slice. The small bowel in the upper abdomen was not included. A sample to illustrate the delineation of the bowels is shown in Figure 1. The dose-volume relationship was calculated in the planning system. The dose was prescribed to the isodose curve (95% to 100%) that surrounded the treatment volume at risk. The onset and grade of diarrhea during whole-pelvic irradiation were recorded as small-bowel toxicity up to 39.6 Gy in 22 fractions.

### 2.3. Treatment Technique

The RT treatment technique typically used opposed posterior to anterior and opposed lateral field arrangement for the whole pelvis [6] to fulfil the homogeneity requirement. External whole-pelvic irradiation was initially administered with photons from a 10 or 15 MV linear accelerator (Varian Medical Systems, Palo Alto, CA) with a four-field technique. The dose per fraction was 1.8 Gy in 5 fractions weekly. The planning dose was 39.6–45 Gy in 22–25 fractions. An additional boost (5.4–9 Gy in 3–5 fractions) was given to the bilateral parametrial and pelvic wall through anteroposterior/posteroanterior ports with a 4 cm central shielding in patients with greater than Stage IIA cervical cancer without hysterectomy. In patients with hysterectomy, the boost doses (5.4–9 Gy in 3–5 fractions) to low pelvis were delivered. The fields and doses beyond 39.6 Gy are shown in Table 1.

### 2.4. Brachytherapy

High-dose-rate intracavitary brachytherapy through a remote after-loading system (microSelectron; Nucletron, Veenendaal, The Netherlands) using 192Ir sources was given. The prescribed dose of brachytherapy was either 4.5 or 6 Gy at Point A and 27 Gy in 6 fractions or 24 Gy in 4 fractions. In vaginal intracavitary brachytherapy, we delivered 8–15 Gy in 2–5 fractions.

### 2.5. Gastrointestinal Toxicity

The severity of acute diarrhea during treatment was graded using the common toxicity criteria (CTC) up to 39.6 Gy in 22 fractions [16]: grade 0 (none), grade 1 (an increase from 2 to 3 stools per day over pretreatment), grade 2 (an increase of 4–6 stools per day or nocturnal stools), grade 3 (increase of >7 stools per day or incontinence or need for parenteral support for dehydration), and grade 4 (physiologic consequences requiring intensive care or hemodynamic collapse).

According to previous studies, which suggested that gastrointestinal drugs may influence bowel symptoms, we managed patients with gastrointestinal symptoms by using a uniform protocol, patients with CTC: grade 1 diarrhea (mebeverine was prescribed), grade 2 diarrhea (loperamide was added), grade 3 diarrhea (loperamide was prescribed three times a day), and grade 4 diarrhea (hospitalization and radiotherapy were interrupted until diarrhea downgraded to grade 2 diarrhea or less).

In this study, we investigated the NTCP predictive models for the incidence of acute diarrhea. The severity of diarrhea was graded into four levels. In our data, there were no patients suffering with grade 4 diarrhea, and we classified grade 2 and greater diarrhea (grade 2^+^) as clinically significant.

### 2.6. Dose-Volume Response Modeling

The cutting endpoints (grade 2^+^ acute diarrhea toxicities) were analyzed using logistic regression NTCP analysis with an extended bootstrapping technique, as described by El Naqa et al. [17] and Beetz et al. [8, 10]. We obtained the range of small-bowel volume in doses from V4 Gy to V40 Gy, at 10% intervals with the checkpoint dose 39.6 Gy (≈40 Gy). The most significant dose-volume predictive factor for the logistic regression model was determined by using the LASSO with bootstrapping technique [8, 10]. The LASSO was first proposed by Tibshirani in 1996; details can be found in [12, 18–20]. It uses the following equation to shrink the coefficients and select the predictive factors:

(1) $$\begin{array}{c} \underset{\beta }{\text{argmin}}{||Y-X\beta ||}^{2}\;\;\text{subject}\;\text{to}\;||\beta ||=\overset{d}{\underset{j=0}{\sum }}\left|\beta_{j}\right|\leq t, \end{array}$$

where *d* is the number of variables selected and *t* is tuning parameter that controls the degree of penalty [12, 21]. To account for the overfitting problem, two datasets were used, that is, a training set and a test set; a model was built based on a training set and fitted to the training set itself and also tested with a test set. We used nested 10-fold cross validation to obtain the optimum predictive factors [12, 14, 19]. The model with medium AUC performance was selected as the optimum model with the most significant dose-volume predictive factor.

Toxicity and dose-volume predictive data were then fit to a logistic NTCP function:

(2) $$\begin{array}{c} \text{NTCP}={\left[1+{\left(\frac{\text{T}{\text{V}}_{50}}{V}\right)}^{4\gamma }\right]}^{-1}, \end{array}$$

where *V* is the volume of small bowel receiving a given dose level, TV_50_ is the tolerance volume corresponding to 50% incidence of complications, and *γ* is the normalized slope of the volume response curve. The best-fitting values of model parameters were determined using maximum likelihood analysis and the 95% confidence intervals were found using the profile likelihood method.

The system performance and calibration were evaluated by the AUC, Brier score, *R* ^2^, Omnibus test, and Hosmer-Lemeshow test [8, 9]. Statistical analyses were performed using SPSS 19.0 (SPSS, Chicago, IL).

## 3. Results

Ninety-five patients were included in the analysis. We classified patients into two groups. Patients without prior abdominal surgery were named group 0 (*n* = 61). They all had cervical cancer. Those who had undergone prior abdominal surgery were named group 1 (*n* = 34). Their diseases included cervical cancer, endometrial cancer, and uterine/adnexa sarcoma. During radiotherapy, seven patients (11%), thirty-one (51%), seventeen (28%), and six (10%) patients in group 0 had grades 0, 1, 2, and 3 diarrhea, respectively. Eight patients (23.5%), nine (26.5%), nine (26.5%), and eight (23.5%) patients in group 1 had grades 0, 1, 2, and 3 diarrhea, respectively. No patients suffered from grade 4 diarrhea. The number of patients experiencing grade 2^+^ acute diarrhea toxicity was 23/61 (38%) in the group without abdominal surgery and 17/34 (50%) in those with abdominal surgery.

**Figure 2:**
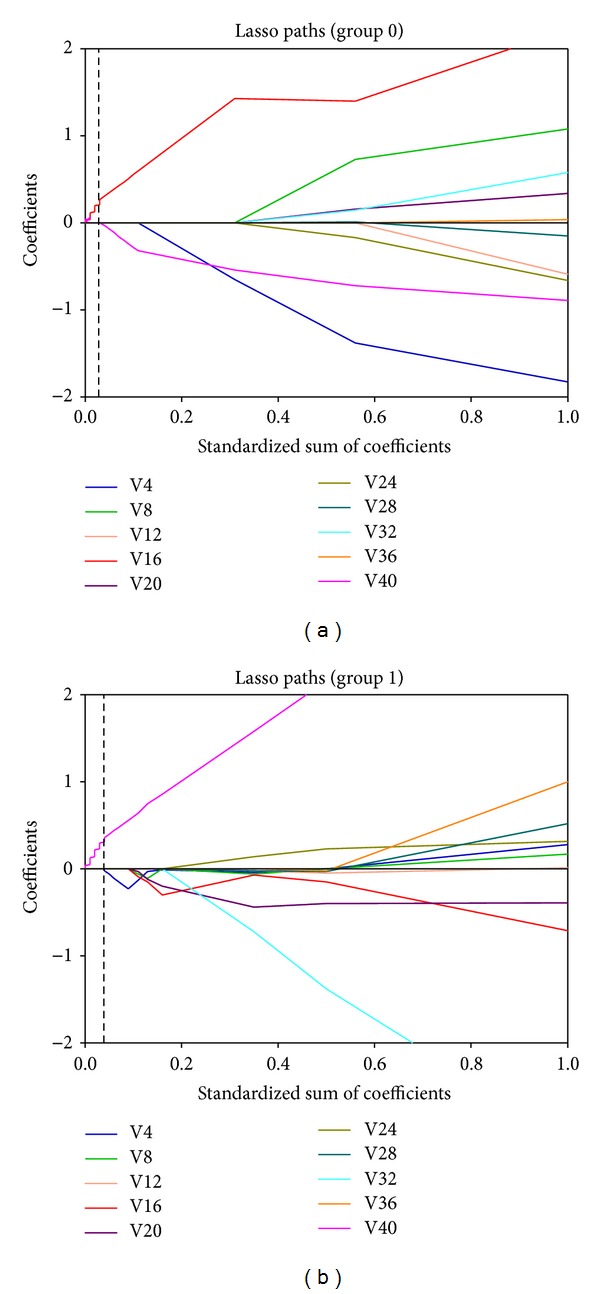
The LASSO shrinking path diagrams for the dosimetric candidate predictive factors in group 0 (a) and group 1 (b), respectively. LASSO: least absolute shrinkage and selection operator; group 0: patients without prior abdominal surgery; group 1: patients with prior abdominal surgery; V4~V40: the range of small-bowel volume in *x* Gy of dose.

The most significant dose-volume predictive factor for the logistic regression NTCP model was determined by using the LASSO with bootstrapping technique (the LASSO shrinking path diagrams are shown in Figure 2). We used 300 bootstraps for each analysis. The initial dosimetric candidate predictive factors were shown in Table 2. The most significant predictor was found for the logistic regression NTCP model with V16 Gy as the cutoff dose for group 0 and V40 Gy for group 1, respectively.

**Figure 3:**
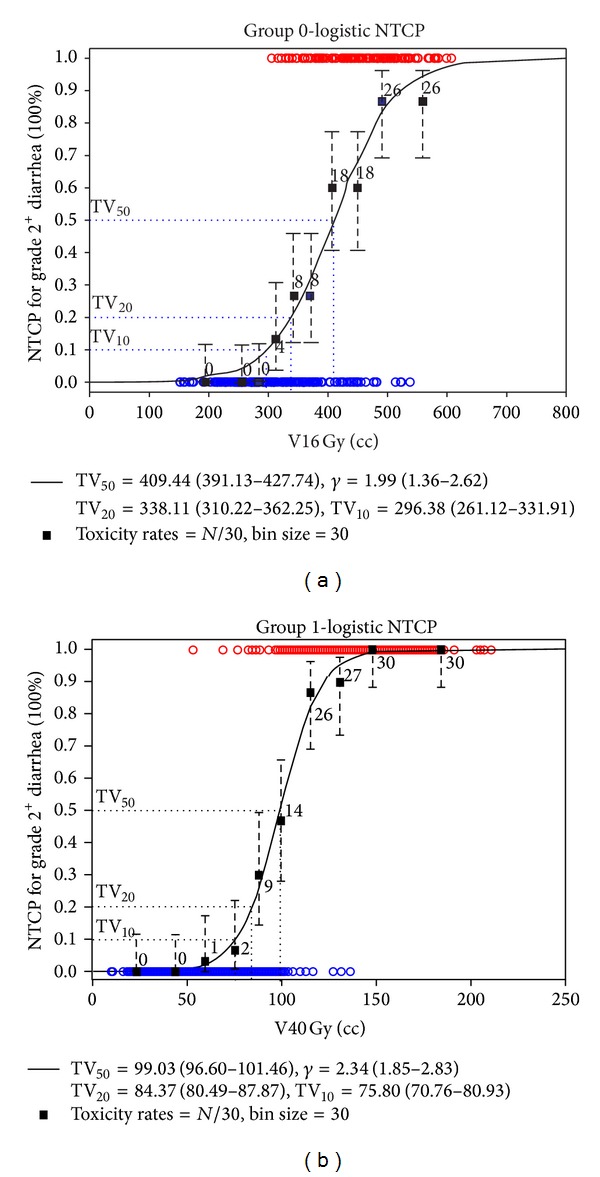
The logistic normal tissue complication probability model with V16 Gy as the cutoff dose for group 0 (a) and V40 Gy for group 1 (b). TV_50_ is the tolerance volume corresponding to 50% incidence of complications, and *γ* is the normalized slope of the volume response curve.

The fitted dose-response curves (logistic NTCP model) for the incidence of grade 2^+^ acute diarrhea toxicity for the gynecological patients with/without prior abdominal surgery are shown in Figure 3. NTCP fitted parameters were TV_50_ = 409.4 cc (CI: 391.1–427.7 cc), *γ* = 1.92 (CI: 1.36–2.62) and TV_50_ = 99.0 cc (CI: 96.6–101.5 cc), *γ* = 2.34 (CI: 1.85–2.83) for the patients in groups 0 and 1, respectively. And TV_10_ ≈ 290 cc for V16 Gy; TV_10_ ≈ 75 cc for V40 Gy, respectively (TV_10_ is the tolerance volume corresponding to 10% incidence of complications).

**Figure 4:**
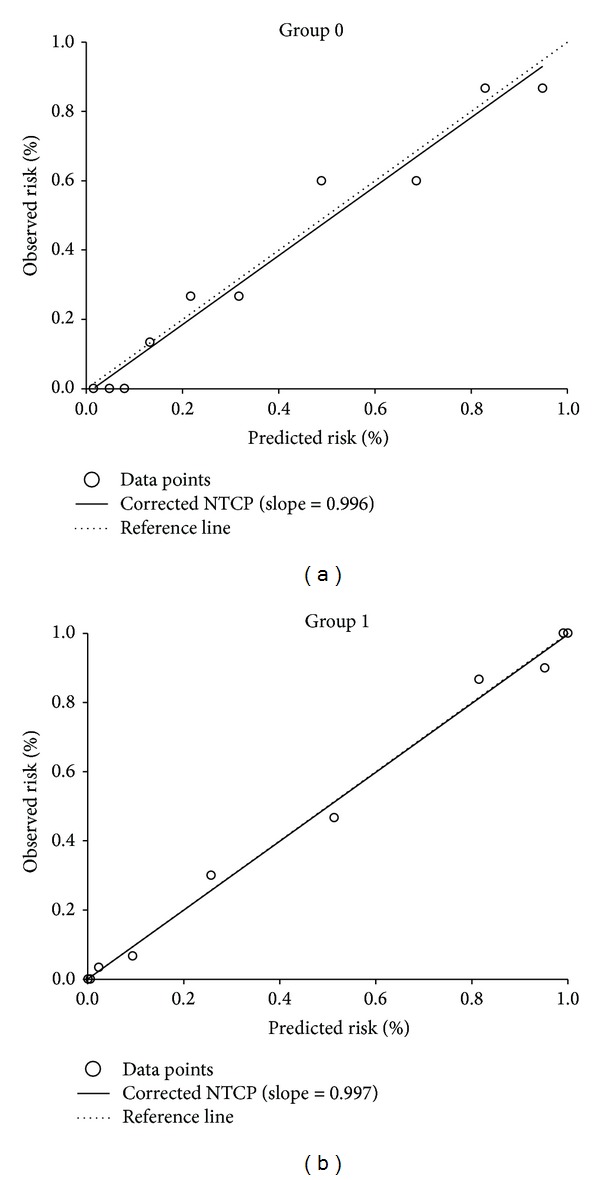
Calibration of a predictive model for patients in group 0 (a) and for patients in group 1 (b), respectively.

The overall performance and calibration for the NTCP models were satisfactory and corresponded well with the expected values (Table 3). The AUC for the optimal model was 0.90 (range 0.79–0.95) and 0.96 (range 0.91–0.99) in patients without and with previous abdominal surgery, respectively. Finally, the calibration slope of ≥0.99 for both models showed a significant agreement between predicted risk and observed outcome for both LASSO NTCP models (Figure 4).

## 4. Discussions

Many researches have discussed the different dose population of gastrointestinal tract between preoperative and postoperative radiotherapy. Shadad et al. thought that previous abdominal surgery increases the risk of radiation toxicity because anatomical changes that increase intestinal exposure to radiation such as postoperative small intestine prolapse into the pelvic cavity or surgical adhesions that fix intestinal segments within the radiation field can all predispose part of the intestine to receive higher doses of radiation [22]. Robertson et al. [5] noted that the incidence of grade 3 diarrhea is higher in rectal cancer patients with abdominal surgery than in those without abdominal surgery, with rates of 28% and 18%, respectively. Recently, they reported a similar result; this group found that the incidences of diarrhea in patients with and without prior abdominal surgery were 29% and 14%, respectively [23]. The corresponding rates were 23% and 10% in the present study, showing a lower incidence of grade 3 diarrhea. The reason for this may be related to different cancers. No rectal disease was noted in our patients. However, rectal function impairments were noted in patients with rectal mass (preoperative) or rectal resection (postoperative) [23]. Therefore, using CTC grading, patients with rectal cancer may have more severe diarrhea than our gynecological patients. Furthermore, we noted no larger V4–V40 Gy in group 1 than group 0 patients (Table 2). However, more patients developed grade 2-3 diarrhea. Therefore, we confirmed the importance of abdominal surgery on acute diarrhea.

Although patients in group 0 had larger small-bowel volumes irradiated than patients in group 1. (Table 2), the diarrhea rate was higher in group 1 patients. This points out the importance of abdominal surgery on dosimetry and toxicity of the small bowel.

Group 0 patients had an older age than those in group 1 (Table 1). Based on our prior study [24], small-bowel volumes are larger than in elder patients. We do not know the cause of this phenomenon. In patients with an intact uterus, atrophic uterine may mimic hysterectomy and also increases the volume of small bowel irradiated. Therefore, the old age effect may explain why group 0 patients had larger small-bowel volume than group 1 patients. Because elder patients account for the majority of patients undergoing definitive radiotherapy, their small-bowel volume may enlarge and dose constraints (V40 Gy < 75 cc or V16 Gy < 290 cc) are very important for using IMRT.

The most significant dose-volume predictive factor for the logistic regression NTCP model can be determined by using statistical analysis. Many researchers used the log likelihood (LL), average likelihood, stepwise selection, Bayesian information criterion (BIC), and Akaike information criterion (AIC) to deal with this topic [8]. Xu et al. [12] showed that their NTCP models for xerostomia developed with LASSO have considerably better prediction performance than the commonly used stepwise selection method. Early NTCP models, like the LKB [25] and the univariate logistic regression model [26, 27], are based on information derived from dose-volume histograms generated from dose distributions in the target volumes and the surrounding organs that are at risk. If the models developed using V4–V40 Gy individually did not take into account abdominal surgery; the AUC values for the model were below 0.65. We considered the importance of abdominal surgery on acute diarrhea and quantified the relationship between the incidence of acute diarrhea and dose-volume effects of the small bowel. The most significant dose-volume predictive factor for the logistic regression NTCP model was determined by the LASSO with V16 Gy as the cutoff dose for group 0 and V40 Gy for group 1, respectively. The system performance AUC values improved from 0.65 to 0.90 for group 0 and from 0.58 to 0.96 for group 1, respectively. We suggest that this is useful for clinical practice in consideration of time and cost efficiency; overall variable selection and coefficients shrink were performed in one step while handling the multicollinearity problem [12, 19, 28].

Many studies have discussed the dose-volume effects. Baglan et al. reported on 40 patients with rectal cancer treated with the four-field technique. Eight patients (20%) did not receive abdominal surgery before radiotherapy. The small-bowel volume receiving at least 15 Gy (V15 Gy) was the best predictor [4]. Tho et al. noted that small-bowel volume correlated strongly with severity of diarrhea at 15 Gy (V15 Gy) < 100 cc in 41 patients undergoing preoperative chemoradiotherapy for rectal cancer [7]. Several other studies have reported dose-volume relationships for the bowel in patients treated for rectal cancer with four-field techniques [1, 5]. They all found a high correlation of 15 Gy (V15 Gy) as a predictor of small-bowel toxicity. The quantitative analysis of normal tissue effects in the clinic (QUANTEC) review summarizes the available 3D-CRT data to update and refine the normal tissue dose/volume tolerance guidelines for small bowel which is “the absolute volume of small bowel receiving ≥15 Gy should be held to <120 cc when possible to minimize severe acute toxicity <10%, if delineating the contours of bowel loops themselves” [29, 30]. In this study, we found that to keep the incidence of grade 2^+^ acute small-bowel toxicity below 10%, the small-bowel volume above the prescription dose V16 Gy should be maintained at <290 cc for patients without prior abdominal surgery; and the prescription dose V40 Gy should be held at <75 cc for patients with prior abdominal surgery. The difference in volume constraints (<120 cc versus <290 cc) on 15-16 Gy level may be dependent on grade of diarrhea (grade 3^+^ versus grade 2^+^) and rectal cancer (yes versus no), respectively. In addition to small bowel, the rectum is the other organ at risk for acute diarrhea. In rectal cancer patients, diarrhea is sensitive to small volumes (V15 Gy) of the small bowel because of rectal function impairment.

To our knowledge, recently, there has been no standard dose-volume constraint for the volume or percentage of irradiated small bowel in gynecological patients. Therefore, the aim of the present study was to develop specific NTCP values. Huang et al. [6] showed that gynecologic patients without and with abdominal surgery have different volume effects on small-bowel toxicity during whole-pelvic irradiation. Low-dose volumes can be used as a predictive index of grade 2^+^ diarrhea in patients without abdominal surgery. The full-dose volume is more important than low-dose volume for grade 2^+^ diarrhea in patients with abdominal surgery. In this study, we confirmed that and determined the most significant dose-volume predictive factor by using the LASSO with bootstrapping technique. Our finding showed the most significant predictor for the logistic NTCP model with V16 Gy as the cutoff dose for patients without prior abdominal surgery and V40 Gy for patients with prior abdominal surgery. Logistic regression NTCP model parameters were found: approximately TV_50_ = 400 cc for V16 Gy and TV_50_ = 100 cc for V40 Gy, respectively.

The fact that chemotherapy, a nondosimetric patient factor, may affect the risk of acute diarrhea toxicity is an issue of special concern. In this study, during radiotherapy with concurrent chemotherapy, the number of patients experiencing grade 2^+^ acute diarrhea toxicity was 18/46 (39%) in the group without abdominal surgery (group 0) and 11/17 (65%) in those with abdominal surgery (group 1). The incidences of toxicity differences between these two cohorts were presented (*P* = 0.013). Chitapanarux et al. found that grade 1-2 acute and late diarrhea were observed in 20 and 40% caused by chemotherapy, respectively, in their thirty metastatic or recurrent cervical cancer patients [31]. Moore et al. studied patients receiving pelvic RT and showed that seven were treated with weekly cisplatin at doses of 30 and 40 mg/m^2^, with one dose-limiting toxicity, that is, febrile neutropenia with grade 3 diarrhea [32]. Rose et al. reported that eleven patients received sixty-three doses of the topotecan/cisplatin combination therapy, nine with grade 2 diarrhea toxicity during pelvic radiation therapy in patients with locally advanced cervical cancer [33]. These reports show that the use of chemotherapy was typically correlated with acute diarrhea toxicity risk. In addition, no significant difference for the small-bowel volume irradiated was observed in the patient groups with/without concurrent chemotherapy, *P* > 0.52 for group 0 and *P* > 0.28 for group 1, respectively. Further studies may be necessary to evaluate what regimen for chemotherapy against acute diarrhea toxicity is better during RT.

There are a number of potential limitations of this study. van der Schaaf et al. [13] reported that approximately 200 patients are needed to obtain a model with high predictive power. In this study, the number of patients assessed for grade 2^+^ acute diarrhea toxicities was under the recommended 200 patients. Therefore, a larger study sample is required to demonstrate the independent association of these NTCP models with the risk of grade 2^+^ acute diarrhea toxicity. Moreover, treatment methods may differ among nations and institutions. Differences in radiation modality may create different kinds and different levels of acute diarrhea toxicity. The risk of small-bowel acute diarrhea may be influenced by the techniques used for treatment or factors other than dose, such as the baseline patient risk factors, the coirradiation of other organs, or the fact that bowel motion may be needed for further investigation.

## 5. Conclusions

The LASSO NTCP model can be used to predict the risk of grade 2^+^ acute diarrhea toxicity. This result illustrates that gynecologic patients with or without prior abdominal surgery have different volume effects on small-bowel toxicity. We suggest a dose-volume constraint for the volume of bowel that can be irradiated.

## Figures and Tables

**Table 1:** Characteristics of patients.

	Group 0 (*n* = 61) Number (%)	Group 1 (*n* = 34) Number (%)	*P* value
Age (y)			0.031
Mean	60.1	54.9	
Range	29–90	32–75	
<50	16 (26)	9 (26)	
51–60	18 (30)	15 (45)	
61–70	11 (18)	9 (26)	
>70	16 (26)	1 (3)	
BMI			0.360
Mean	24.9	25.7	
Range	16.6–35.1	20.1–34.5	
<20	8 (13)	0 (0)	
20–26	27 (44)	20 (59)	
>26	26 (43)	14 (41)	
Stage			0.007
I-II	52 (85)	19 (56)	
III-IV/recurrent	9 (15)	15 (44)	
Diabetes			0.763
No	52 (85)	30 (88)	
Yes	9 (15)	4 (12)	
Hypertension			0.779
No	50 (82)	29 (85)	
Yes	11 (18)	5 (15)	
Chemoradiotherapy			0.013
No	15 (25)	17 (50)	
Yes	46 (75)	17 (50)	
External beams doses (Gy)			0.469
WP 19.8, PM/LP 32.4	0 (0)	1 (2.9)	
WP 39.6	20 (33)	5 (14.7)	
WP 39.6, PM/LP 45	27 (44)	23 (67.6)	
WP 39.6, LP 50.4	0 (0)	2 (5.9)	
WP 45	4 (7)	1 (2.9)	
WP 45, PM/LP 50 4	10 (16)	2 (5.9)	

Group 0: patients without prior abdominal surgery; group 1: patients with prior abdominal surgery; BMI: body mass index; WP: whole pelvis; LP: low pelvis; PM: parametrium.

Differences between the group 0 and group 1 cohort were described with an independent sample *t*-test for continuous variables and chi-square test for dichotomous variables.

**Table 2:** Dosimetric candidate predictive factors initially.

Volumes	Group 0			Group 1		
	Range	Median	Logistic correlation	Range	Median	Logistic correlation
V4 Gy	76–990	460	−0.009	65–772	329	−0.039
V8 Gy	65–889	422	0.016	39–700	297	0.043
V12 Gy	57–824	385	−0.094	27–665	276	−0.042
V16 Gy	51–772	348	0.166	20–634	255	0.050
V20 Gy	15–721	230	−0.003	13–603	175	−0.021
V24 Gy	6–678	177	−0.001	12–578	135	0.026
V28 Gy	4–651	138	−0.033	10–324	111	−0.023
V32 Gy	3–611	115	0.055	8–296	102	0.069
V36 Gy	1–584	104	0.024	4–274	96	−0.407
V40 Gy	0–545	89	−0.063	1–249	77	0.740

V4~V40 Gy: the range of small-bowel volume in *x* Gy of dose; group 0: patients without prior abdominal surgery; group 1: patients with prior abdominal surgery.

**Table 3:** System performance evaluation.

Group	Cutoff dose	AUC (range)	Accuracy (range)	*R* ^2^	Brier	Slope of calibration curve	HL
0	V16 Gy	0.90 (0.79–0.95)	0.81 (0.70–0.87)	0.98	0.10	0.99	0.14
1	V40 Gy	0.96 (0.91–0.99)	0.90 (0.83–0.93)	0.99	0.07	0.99	0.88

AUC: area under the receiver operating characteristic curve; HL: Hosmer-Lemeshow test.

Group 0: patients without prior abdominal surgery; group 1: patients with prior abdominal surgery.
